# Influence of Carrier Gases on the Quality of Epitaxial Corundum-Structured α-Ga_2_O_3_ Films Grown by Mist Chemical Vapor Deposition Method

**DOI:** 10.3390/ma12223670

**Published:** 2019-11-07

**Authors:** Yu Xu, Chunfu Zhang, Yaolin Cheng, Zhe Li, Ya’nan Cheng, Qian Feng, Dazheng Chen, Jincheng Zhang, Yue Hao

**Affiliations:** Wide Bandgap Semiconductor Technology Disciplines State Key Laboratory, School of Microelectronics, Xidian University, Xi’an 710071, China; xuyuxidian@163.com (Y.X.); chengyaolin96@163.com (Y.C.); zhe_li1024@163.com (Z.L.); qfeng@mail.xidian.edu.cn (Q.F.); dzchen@xidian.edu.cn (D.C.); jchzhang@xidian.edu.cn (J.Z.); yhao@xidian.edu.cn (Y.H.)

**Keywords:** wide-bandgap semiconductor, α-Ga_2_O_3_, mist chemical vapor deposition (mist-CVD), carrier gas, transparent semiconductor

## Abstract

This report systematically investigates the influence of different carrier gases (O_2_, N_2_, and air) on the growth of gallium oxide (Ga_2_O_3_) thin films on c-plane sapphire substrates by using the mist-CVD method. Although XRD and Raman measurements show that the pure corundum-structured α-Ga_2_O_3_ with single (0006) plane orientation was successfully obtained for all three different carrier gases, the crystal quality could be greatly affected by the carrier gas. When O_2_ is used as the carrier gas, the smallest full-width at half maximum (FWHM), the very sharp absorption cutoff edge, the perfect lattice structure, the highest growth rate, and the smooth surface can be obtained for the epitaxial α-Ga_2_O_3_ film as demonstrated by XRD, UV-VIS, TEM, AFM (Atomic Force Microscope), and SEM measurements. It is proposed that the oxygen content in carrier gas should be responsible for all of these results. XPS (X-ray photoelectron spectroscopy) analysis also confirms that more oxygen elements can be included in epitaxial film when O_2_ is used as the carrier gas and thus help improve the crystal quality. The proper carrier gas is essential for the high quality α-Ga_2_O_3_ growth.

## 1. Introduction

As an ultra-wide-bandgap semiconductor with the obvious advantages of stable physical chemistry, low dielectric constant, and high mechanical strength, gallium oxide (Ga_2_O_3_) is attracting increasing attention as a new promising competitor to III-nitrides and SiC for various applications in high-voltage and high-power electronics and ultraviolet optoelectronics [1]. Compared with other oxides such as ZnO (3.24 eV) and In_2_O_3_ (3.6 eV), Ga_2_O_3_ has a larger bandgap energy of approximately 5 eV, which means a shorter absorption cutoff wavelength and a much higher power application. There are a total of five different polytypes (*α*, *β*, *ε*, *δ*, and *γ*) for Ga_2_O_3_. Until now, the most studied polytype was β-Ga_2_O_3_ because it is easy to obtain bulk and film β-Ga_2_O_3_ materials by the conventional crystal growth or epitaxial growth techniques, such as edge-defined film-fed growth, float-zone method, Czochralski method, molecular beam epitaxy, and metal organic chemical vapor deposition. β-Ga_2_O_3_ has a bandgap of 4.8 eV and high Baliga’s figures of merit (FOM) of 3000, which is obviously superior to GaN and SiC. However, β-Ga_2_O_3_ is not the best candidate in various phases for the power application considering the bandgap. Compared to β phase, corundum-structured α-Ga_2_O_3_, another important phase for Ga_2_O_3_, has a wider bandgap of around 5.3 eV which can result in a larger Baliga’s FOM, in theory. Thus, α-Ga_2_O_3_ has great potential for application in power devices. A wider bandgap of about 5.3 eV means that the absorption cutoff wavelength can be shorter than 240 nm and then α-Ga_2_O_3_ is more suitable for ultraviolet optoelectronics. Although α-Ga_2_O_3_ has great application potential, the research of α-Ga_2_O_3_ still lags far behind β-Ga_2_O_3_ in large part because it is more difficult to obtain the high-quality α-Ga_2_O_3_ material than β-Ga_2_O_3_.

α-Ga_2_O_3_ is a metastable phase and the bulk material still cannot be obtained. There is no commercial α-Ga_2_O_3_ bulk substrate to date, so its homo-epitaxial growth is still difficult. Fortunately, the heterogeneous epitaxy provides an efficient way to obtain the α-Ga_2_O_3_ material. Corundum-structured gallium oxide belongs to the space group of R-3c with the lattice parameters a = b = 4.98 Å, c = 13.43 Å, α = β = 90°, and γ = 120° [2], and the lattice mismatches between α-Ga_2_O_3_ and α-Al_2_O_3_ (sapphire) are only 4.81% and 3.54% in the a- and c-axis directions. The same crystal structure and small lattice mismatch make it easy to grow the α-Ga_2_O_3_ material on the α-Al_2_O_3_ sapphire substrate. More importantly, the same crystal structure means that it is attractive from the viewpoint of fabricating alloys for α-Ga_2_O_3_ with other corundum-structured materials, such as α-Al_2_O_3_, Fe_2_O_3_, and Cr_2_O_3_ for bandgap and material engineering [3], which is another advantage of α- Ga_2_O_3_.

Recent studies have shown that the growth of crystalline α-Ga_2_O_3_ on an inexpensive sapphire substrate is an efficient way to obtain the α-Ga_2_O_3_ material. The key technology for the growth of α-Ga_2_O_3_ is ultrasonic mist chemical vapor deposition (mist-CVD) method [4,5,6]. In the growth of a metal oxide, water solutions of safe and inexpensive chemicals containing the metal, for example, acetate or acetylacetonate, have been used as the source. By atomizing the source solution ultrasonically, it turns into mist particles, which are then transferred by a carrier gas to a reaction chamber. In this way, metal elements are supplied without the use of organometallic sources. They react with an oxygen source, which may be water or oxygen gas. This offers sufficient overpressure of oxygen with respect to the metal source and prevents the formation of oxygen vacancies. Therefore, mist-CVD method is suitable for epitaxial α-Ga_2_O_3_ on sapphire substrate and can reduce the material cost [7,8]. Based on the grown α-Ga_2_O_3_, various applications of Metal Epitaxial-Semiconductor Field Effect Transistor [9], Schottky barrier diodes [10], and solar-blind photodetectors [11] have been demonstrated. For example, a high performance Schottky diode with a breakdown voltage over 1 kV and a small specific on-resistance of 2.5 mΩ·cm^2^ has been achieved and a normally-off MOSFET has been shown based on α-Ga_2_O_3_ material grown by a mist-CVD system [9,10]. However, the present device performance is greatly inferior to the β-Ga_2_O_3_ counterparts and the main reason is still the poor α-Ga_2_O_3_ film quality. Thus, more attention is urgently required to improve the quality of α-Ga_2_O_3_ now and in the future. Recently, highly crystalline α-Ga_2_O_3_ thin films have been successfully grown at atmospheric pressure by mist-CVD on c-sapphire substrates, whose temperatures of 400–500 °C are reasonably low and the optimal growth conditions of solution concentration, growth temperature, carrier gas velocity, and film thickness have also been investigated [12,13]. However, there is still no systematical study about how the different carrier gas affects the film quality.

In this paper, we systematically investigate the influence of different carrier gases (O_2_, N_2_, and air) on the film quality for the growth of α-Ga_2_O_3_ on c-plane sapphire substrates by using the mist-CVD method. It is demonstrated that the crystallization quality will be different when the gallium source is carried by different gases. When N_2_ and O_2_ are used as the carrier gases, α-Ga_2_O_3_ achieves a relative smooth surface. When O_2_ is the carrier gas, α-Ga_2_O_3_ achieves the smallest half-height width. The oxygen element in the carrier gas may be an important reason to prevent the generation of oxygen vacancies, thus influencing the quality of the thin films. The results provide constructive perspectives for the material quality improvement.

## 2. Materials and Methods

In the present experiment, we used gallium acetylacetonate as the gallium source, which was dissolved in deionized water. A small quantity of hydrochloric acid was added to dissolve gallium acetylacetonate completely. The concentration of the solution was adjusted to 0.05 M. By atomizing the source solution ultrasonically, it turned into mist particles (diameter of ~3 µm at an ultrasonic frequency of 2.4 MHz), which was then carried by air, N_2_, and O_2_, respectively, to the heated reaction chamber. In the chamber, the (0001) sapphire substrate was placed on a sample holder that was kept at 400 °C, because under this condition a high quality α-Ga_2_O_3_ can be obtained, as shown in Figure S1 in the supplemental information. The growth time was kept at 1h and the rate of the carrier gas was set to be 6 L/min.

The structural properties of Ga_2_O_3_ films were investigated using a range of complementary techniques. X-ray diffraction (XRD) patterns were obtained from an X-ray diffractometer (D8 Advance, Bruker, Karlsruhe, Germany). The transmittance was measured by a dual-beam 950 UV-VIS spectrometer. XPS measurements were performed by the Escalab 250Xi (Waltham, MA, USA) with a source of monochromatic Al-Ka (1486.6 eV). The film morphologies were characterized by a field emission scanning electron microscope (SEM JSM-7800F, Tokyo, Japan), atomic force microscopy (AFM) (Agilent 5500, Palo Alto, Santa Clara, CA, USA), and high-resolution transmission electron microscopy (TEM) (Tecnai G2 F20 S-Twin, Hillsboro, OR, USA). The Raman spectra were measured using a confocal Jobin Yvon LavRam HR800 micro-Raman spectrometer (Edison, NJ, USA) with a charge-coupled device (CCD) detector.

## 3. Results

**Figure 1:**
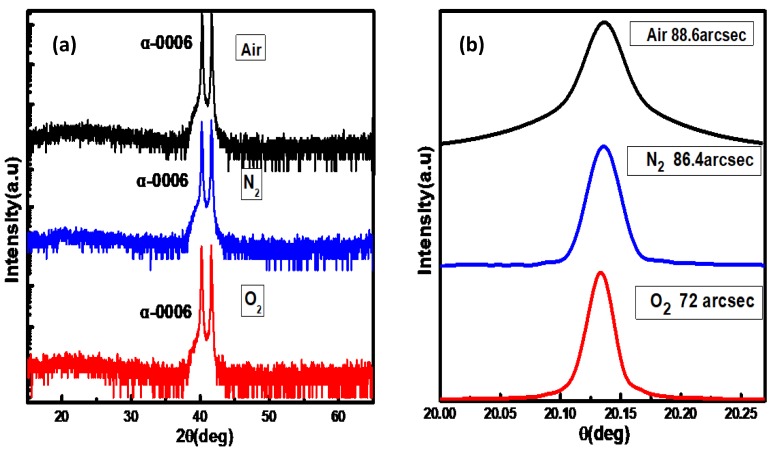
(**a**) X-ray diffraction 2θ/θ scan spectra for Ga_2_O_3_ films grown with different carrier gases. (**b**) The corresponding (0006) XRD diffraction rocking curves. The full-width at half maximum of Ga_2_O_3_ films grown with air, N_2_, and O_2_ as the carrier gases are 88.6 arcsec, 86.4 arcsec, and 72 arcsec, respectively.

Figure 1 shows the XRD spectra of the samples with air, N_2_, and O_2_ as the carrier gases. The spectra are dominated by the diffraction peaks at 40.26° and 41.66°, which correspond to the (0006) planes of α-Ga_2_O_3_ epilayer and sapphire substrate, respectively. No other peaks are found, which shows that all of the Ga_2_O_3_ films show the obvious pure alpha phase. These XRD spectra show that the α-Ga_2_O_3_ films had a preferential c-axis orientation along the c-axis of the sapphire substrate and the calculated lattice constant along the c-axis is 1.34 nm. Paying attention to the sample grown with O_2_ as the carrier gas, the full-width at half maximum (FWHM) of the ω scan rocking curve is as small as 72 arcsec, indicating a high quality α-Ga_2_O_3_. However, for the samples grown with air and N_2_ as the carrier gases, FWHM of the ω scan rocking curves are 88.6 arcsec and 86.4 arcsec, respectively, indicating a relatively inferior crystal quality. Considering the different oxygen content in the carrier gases, it is supposed that Ga_2_O_3_ film grown with O_2_ as the carrier gas has less defects, such as oxygen vacancies (V_O_), which may be the key to improving the quality of crystallization.

**Figure 2:**
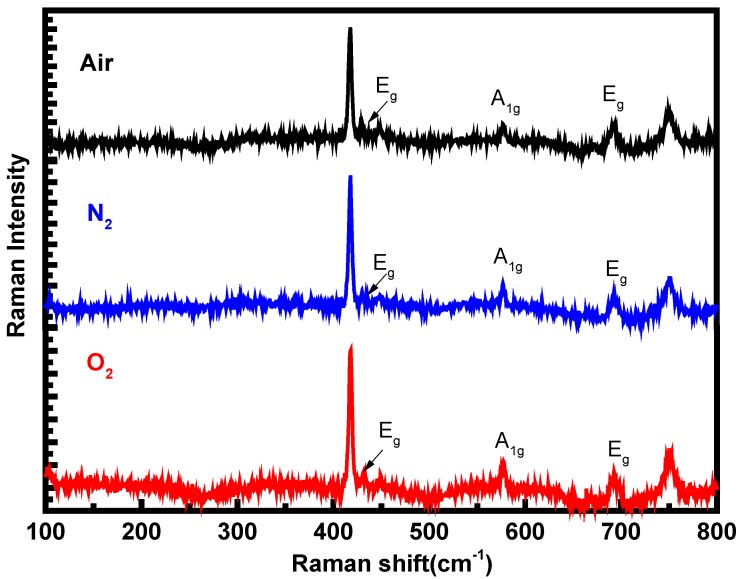
Raman spectra of the α-Ga_2_O_3_ epilayers grown with air, N_2_, and O_2_ as the carrier gases.

Raman spectra of the epilayers grown with air, N_2_, and O_2_ as the carrier gases were measured between 100 cm^−1^ and 800 cm^−1^ at room temperature to confirm the crystalline quality of the deposited films, and the results are presented in Figure 2. A 514 nm laser was used as the excitation source and the laser beam was focused by a microscope lens system (×50 ulwd) yielding a spot size of 1 μm during the Raman measurement. The Raman peaks at 418 cm^−1^ and 749 cm^−1^ belong to the sapphire substrate [14]. The Raman peaks located at 431.3 cm^−1^, 577 cm^−1^, and 692 cm^−1^ are the Raman-allowed vibrational modes of E_g_, A_1g_, and E_g_ for α-Ga_2_O_3_, respectively, and are consistent with the theoretical calculations [15]. The high-frequency A_1g_ mode at 577 cm^−1^ mainly involves the vibration of oxygen atoms perpendicular to the c-axis. The linewidth of the peak A_1g_ is as narrow as 3.9 cm^−1^ for the epilayer grown with O_2_ as the carrier gas, strongly suggesting the high crystallinity of the epilayer. By comparing the epilayers grown with different carrier gases, the displacement and intensity of the Raman peaks do not change obviously, demonstrating that the stress in the films is mainly determined by other factors, such as lattice mismatch or growth temperature, instead of the carrier gases. It could also be observed that except the Raman peaks of α-Ga_2_O_3_, no other peaks are observed in the Raman spectra, which indicates that all of the epilayers are pure α-Ga_2_O_3_ without other phases and confirm the conclusion from the XRD measurements.

**Figure 3:**
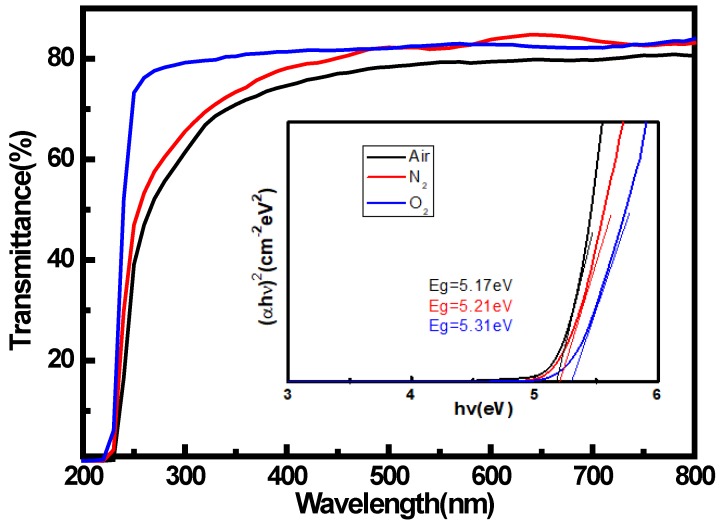
Optical transmission spectra and (αhv)^2^-hv plots of α-Ga_2_O_3_ films.

The variation of optical transmittance spectra (200–800 nm) were performed on α-Ga_2_O_3_ films as shown in Figure 3. All the samples exhibited a transmittance higher than 80% in the visible to near-UV regions. The relationship between the absorption coefficient α and the optical bandgap (E_g_) is αhν = A(hν−Eg)^1/2^, where A is the material-dependent constant, h is the Planck’s constant, and ν is the frequency of the incident light [16]. The optical band gap can be evaluated from the (αhν)^2^ versus photon energy (hν) graph by linear extrapolations to zero absorption coefficient [17]. The inset shows the plot of (αhν)^2^ as function of photon-energy hν. The bandgaps of the obtained materials remained at 5.1–5.3 eV, which is obviously larger than that of β-Ga_2_O_3_. The larger bandgap than that of β-Ga_2_O_3_ is obviously attributed to the crystal structure of α-Ga_2_O_3_ being different from β-Ga_2_O_3_. We note that the α-Ga_2_O_3_ sample grown with O_2_ as the carrier gas is dropped more abruptly than those of air and N_2_ in ultraviolet region. This phenomenon may be caused by less defect in α-Ga_2_O_3_ samples carried by O_2_. During the film growth, extra oxygen will ensure the ideal ratio of O to Ga atomic, which results in higher crystalline quality and leads to its bandgap being close to the ideal value of 5.3 eV. The higher crystalline quality for the sample with O_2_ as the carrier gas is consistent with the XRD measurement results.

**Figure 4:**
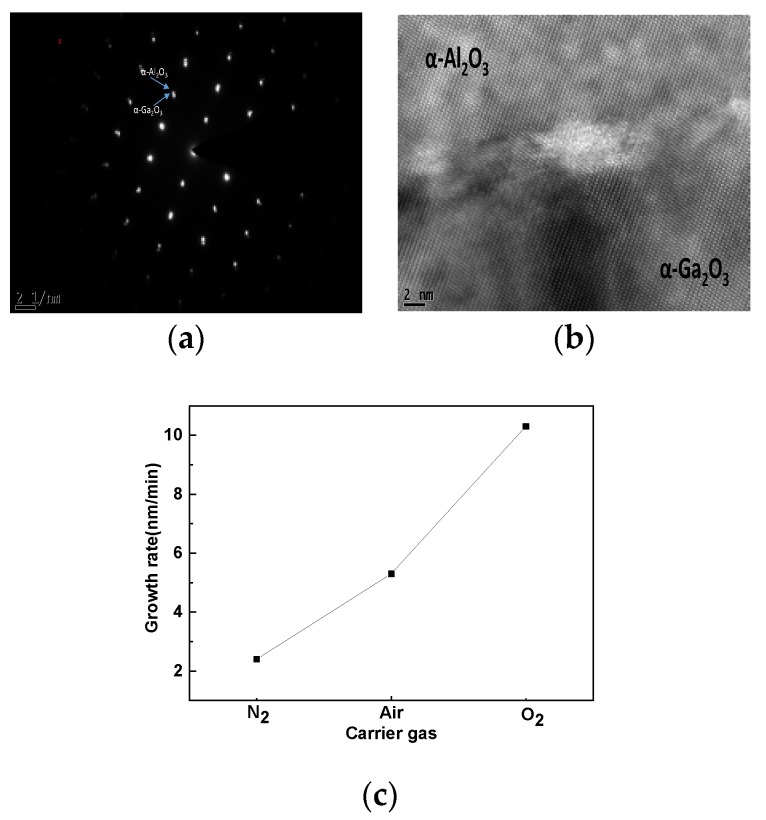
TEM images. (**a**) Diffraction spots of α-Ga_2_O_3_/α-Al_2_O_3_ for the sample with O_2_ as the carrier gas, (**b**) cross-sectional α-Ga_2_O_3_/α-Al_2_O_3_ interface for the sample with O_2_ as the carrier gas. (**c**) Growth rate of α-Ga_2_O_3_ films grown by air, N_2_ and O_2_ carrier gas.

Figure 4a shows the electron diffraction patterns of the Ga_2_O_3_/Al_2_O_3_ interface for the sample with O_2_ as the carrier gas. The diffraction patterns of both the Ga_2_O_3_ film and the Al_2_O_3_ substrate are rectangular, corresponding to the corundum-structure. All of the diffraction spots of α-Ga_2_O_3_ are situated almost in the α-Al_2_O_3_ spots. The cross-sectional HR-TEM image at the α-Ga_2_O_3_/α-Al_2_O_3_ interface is shown in Figure 4b. As a result of the in-plane strain, the α-Ga_2_O_3_/α-Al_2_O_3_ interface is unclearly identified. At the α-Ga_2_O_3_/α-Al_2_O_3_ interface, we can observe a dark area and this is induced by the in-plane compressive strain in the α-Ga_2_O_3_ layer because the lattice constant of α-Ga_2_O_3_ is larger than that of α-Al_2_O_3_ substrate. The TEM images for the samples, with air and N_2_ as the carrier gases in Figures S1 and S2 also demonstrate the similar electron diffraction patterns and obvious HR-TEM lattice structures, indicating that all of the samples achieve a pure α-Ga_2_O_3_ phase and confirm the conclusion from the XRD measurements again. By comparing the HR-TEM images for the three different samples, the samples with O_2_ and air as the carrier gases show a more complete lattice structure than the sample with N_2_ as the carrier gas, which shows that the adequate efficient oxygen content is essential for the high quality film growth and this result is the same with that from the XRD measurement. The α-Ga_2_O_3_ films deposited on sapphire substrates obtain the thicknesses of 619 nm, 318 nm, and 146 nm for the samples with O_2_, air, and N_2_ as the carrier gases, respectively. These thicknesses correspond to the different growth rates of 10.3 nm/min, 5.3 nm/min, and 2.4 nm/min as shown in Figure 4c. Variable-angle Spectral Ellipsometry (SE) measurements were performed at room temperature in ambient atmosphere with an electronically controlled rotating compensator and Glan Taylor polarizers (J. A. Woollam Co., Lincoln, NE, USA). The Cauchy model was used to fit the thickness of Ga_2_O_3_ thin film. Measurements were carried out at three different incidence angles of 55°, 65°, and 75° over the 193–1000 nm wavelength range. Considering the different oxygen contents in the carrier gases, it can be concluded that more oxygen in the carrier gas can promote the growth rate greatly, and at the same time it can also guarantee the crystal quality as demonstrated by the XRD, UV-VIS and TEM measurements.

**Figure 5:**
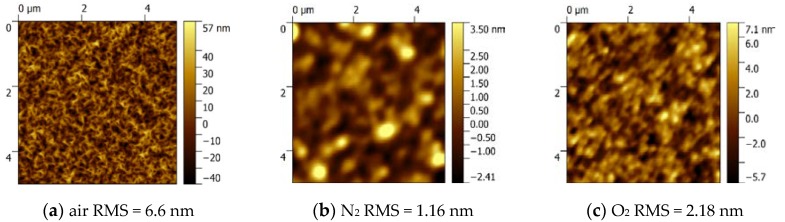

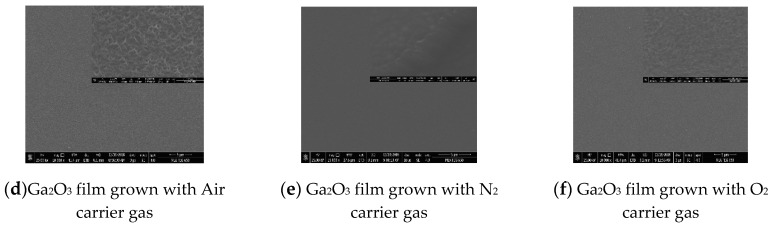
AFM surface images of Ga_2_O_3_ films grown with different carrier gases: (**a**) air (**b**) N_2_, and (**c**) O_2_. SEM pictures for the Ga_2_O_3_ films grown with different carrier gases: (**d**) air (**e**) N_2_, and (**f**) O_2_.

Figure 5 shows the AFM images for the α-Ga_2_O_3_ films prepared by different carrier gases of air (a), N_2_ (b), and O_2_(c). The films have root-mean-square (RMS) surface roughness values of 6.6 nm, 1.16 nm and 2.18 nm, respectively, measured over an area of 5 × 5 μm^2^. The surface morphology for the sample grown with air as the carrier gas is rough and composed of irregular stripes, while the surface morphologies of α-Ga_2_O_3_ thin films grown with N_2_ and O_2_ as the carrier gases are much smoother. It is inferred that the complex components in air induced this rough surface, and the reason behind that requires more research in the future. On the contrary, the pure carrier gas will lead to a much smoother surface. Comparing the sample grown with O_2_ as the carrier gas to the sample with N_2_ as the carrier gas, although the growth rate is improved by about five times, the difference between their RMS values is relatively small. This means that the rapid growth rate with O_2_ as the carrier gas will not degrade the surface morphology. To further investigate the microstructure, we measured SEM images of all α-Ga_2_O_3_ films as shown in Figure 5d–f. As demonstrated in the enlarged pictures, it can be seen that the film grown with air as the carrier gas has indeed a rough surface, and the other samples show the relatively smooth surface, which confirm the results from the AFM measurement.

**Figure 6:**
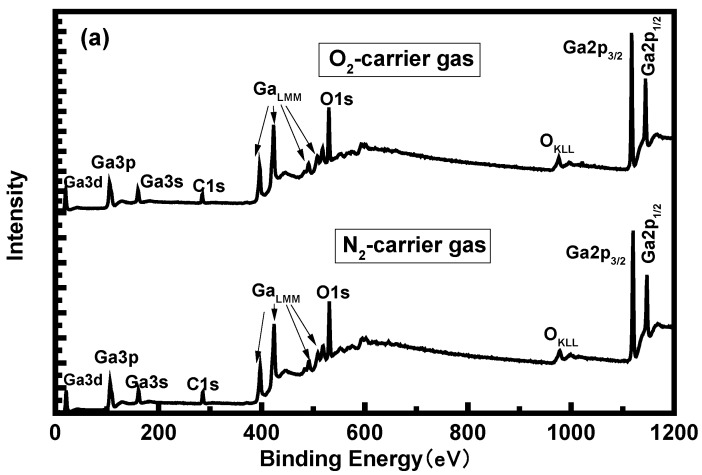

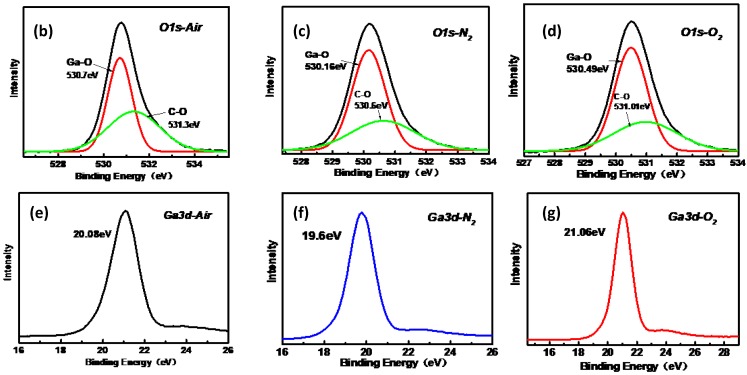
(**a**) X-ray photoelectron wide spectra for the α-Ga_2_O_3_ sample grown with O_2_ and N_2_ as the carrier gas. X-ray photoelectron spectra of O 1*s* peaks for the samples grown with air (**b**), N_2_ (**c**), and O_2_ (**d**) as the carrier gases. X-ray photoelectron spectra of Ga 3*d* peaks for the samples grown with air (**e**), N_2_ (**f**), and O_2_ (**g**) as the carrier gases.

To assess the elemental composition of the α-Ga_2_O_3_ films, XPS measurements were conducted for the epitaxial films on sapphire. The result for the sample with O_2_ as the carrier gas is presented in Figure 6a and only three elements (C, Ga, and O) are observed in the films. The position of the Ga 2*p*_3/2_ and Ga 3*d* binding energy peaks confirms the presence of Ga_2_O_3_ [18]. It can be observed from the spectra that the Ga2*p*_3/2_ and Ga2*p*_1/2_ signal peaks are located at 1118.5 eV and 1145.5 eV respectively, The energy difference between the two signal peaks is about 27 eV, which is consistent with the Ga2*p* signal peak energy difference reported in the literature [19]. The binding energy position of Ga3*d* signal is 21.05 eV, which is consistent with the results reported in the literature [20]. The result for the samples with air as the carrier gases is shown in Figures S3 and S4. The same peak positions appear in both samples and no significant peak shift and peak intensity changes are observed, indicating the same pure α-Ga_2_O_3_ and correspond to the XRD and Raman results. Figure 6b–d show the peaks of O 1*s* signal around 530.6 eV for the three samples. O 1s signal peaks do not show Gaussian symmetry so Gaussian fitting was carried out for the peaks. It is found that the strong O 1*s* signal peaks appear at 530.6 eV and 532.1 eV. The peak at 530.6 eV originated from the oxygen element in Ga–O bond, while the peak at 532.1 eV originated from the adsorption O on the sample surface [21]. Figure 5e–f show Ga 3*d* spectra. The chemical compositions of the surfaces were determined from the area of the Ga 3*d*, O 1*s*, and C 1*s* peaks taking into account the corresponding sensitivity factors [22]. The calculated atomic ratios of O to Ga for the samples grown with air, N_2_, and O_2_ as the carrier gases were 1.53, 1.51, and 1.56, respectively. It reveals that more oxygen present in the carrier gas can slightly increase the O content of the film, which confirms the guess in the XRD measurement.

We also compared the quality of the same thickness and growth rate of the α-Ga_2_O_3_ films grown by different carrier gases. As shown in Figure S2 in the supplemental information, the growth rate of the α-Ga_2_O_3_ films was set to 10 nm/min for all samples. In Figure S3 in the supplemental information, the thicknesses of all α-Ga_2_O_3_ samples were 500 nm. The optical transmittance spectra show that when O_2_ is the carrier gas, the absorption edge is closer to 234 nm of α-Ga_2_O_3_ and the downward trend of the absorption edge is more obvious. From the AFM images, we assume that with the same growth rate and film thickness, the complex components in air induces rough surface and the pure carrier gas will lead to a much smoother surface. These results fully demonstrate that the partial pressure of oxygen in the carrier gas component can improve the quality of the film, consistent with the above results.

## 4. Conclusions

In this work, we systematically investigated the influence of different carrier gases (O_2_, N_2_, and air) on the grown film quality on c-plane sapphire substrates by using the mist-CVD method. Although XRD and Raman measurements show that the pure corundum-structured α-Ga_2_O_3_ was successfully obtained on the c-plane sapphire substrates with single (0006) plane orientation for all three different carrier gases, the crystal quality could be greatly affected by the carrier gas. When O_2_ was used as the carrier gas, the sample showed the smallest FWHM of about 72 arcsec obtained from the XRD rocking curves. The UV-VIS measurement also showed that the sample grown with O_2_ as the carrier gas showed a very sharp absorption cutoff edge. It is proposed that the oxygen content in carrier gas should be responsible for all of these results. More oxygen in the carrier gas can greatly increase growth rate, and at the same time guarantee the film quality. This is further verified by the smoother surface and rapid growth rate (10.3 nm/min) for O_2_ as the carrier gas compared to air and N_2_ as the carrier gases (5.3 nm/min and 2.4 nm/min respectively). XPS analysis confirmed that more oxygen element can be included in epitaxial film and thus help improve the crystal quality. The proper carrier gas is essential for high quality α-Ga_2_O_3_ growth.

## Supplementary Materials

The following are available online at https://www.mdpi.com/1996-1944/12/22/3670/s1, Figure S1: TEM images of the sample grown with air as the carrier gas. (1) Cross-sectional α-Ga_2_O_3_/α-Al_2_O_3_ interface, (2) diffraction spots of α-Ga_2_O_3_/α-Al_2_O_3_, Figure S2: TEM images of the sample grown with N_2_ as the carrier gas. (1) Cross-sectional α-Ga_2_O_3_/α-Al_2_O_3_ interface, (2) diffraction spots of α-Ga_2_O_3_/α-Al_2_O_3_, Figure S3: X-ray photoelectron wide spectra for the α-Ga_2_O_3_ sample grown with air as the carrier gas, Figure S4: X-ray photoelectron wide spectra for the α-Ga_2_O_3_ sample grown with N_2_ as the carrier gas.
