# Supplementary material for: Influence of Carrier Gases on the Quality of Epitaxial Corundum-Structured α-Ga2O3 Films Grown by Mist Chemical Vapor Deposition Method
Source: Materials (Basel). 2019 Nov 7;12(22):3670. doi: 10.3390/ma12223670 (PMC6888560; doi:10.3390/ma12223670)
Supplement: Supplementary file 1 [file materials-12-03670-s001.pdf]

materials

Supplymentary Materials: Influence of carrier gases on the quality of epitaxial corundum-structured α-Ga_2O_3 films by mist chemical vapor deposition method

Yu Xu, Chunfu Zhang *, Yaolin Cheng, Zhe Li, Ya'nan Cheng, Qian Feng, Dazheng Chen, Jincheng Zhang and Yue Hao

Wide Bandgap Semiconductor Technology Disciplines State Key Laboratory, School of Microelectronics, Xidian University, Xi'an 710071, China; xuyuxidian@163.com (Y.X.); chengyaolin96@163.com (Y.C.); zhe_li1024@163.com (Z.L.); yanancheng@stu.xidian.edu.cn (Y.C.); qfeng@mail.xidian.edu.cn (Q.F.); dzchen@xidian.edu.cn (D.C.); jchzhang@xidian.edu.cn (J.Z.); yhao@xidian.edu.cn (Y.H.)
* Correspondence: cfzhang@xidian.edu.cn

(1)

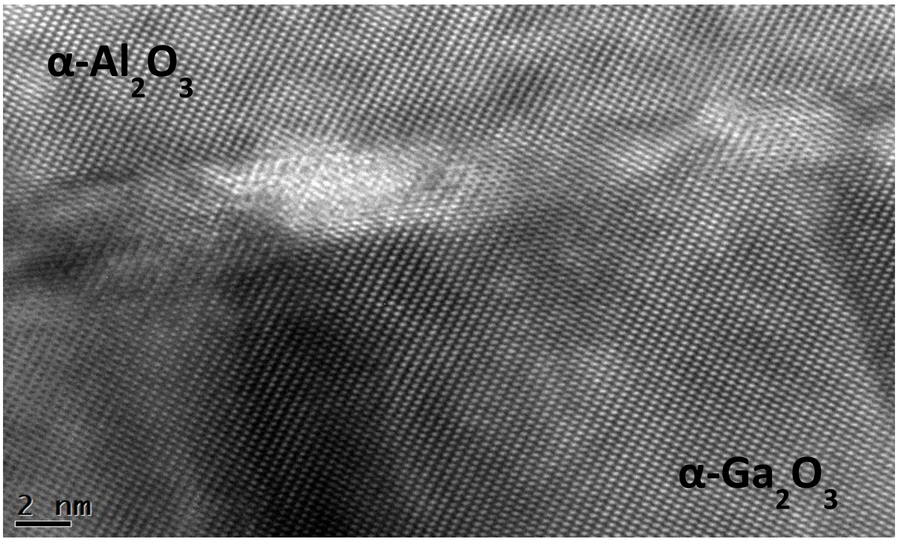

(2)

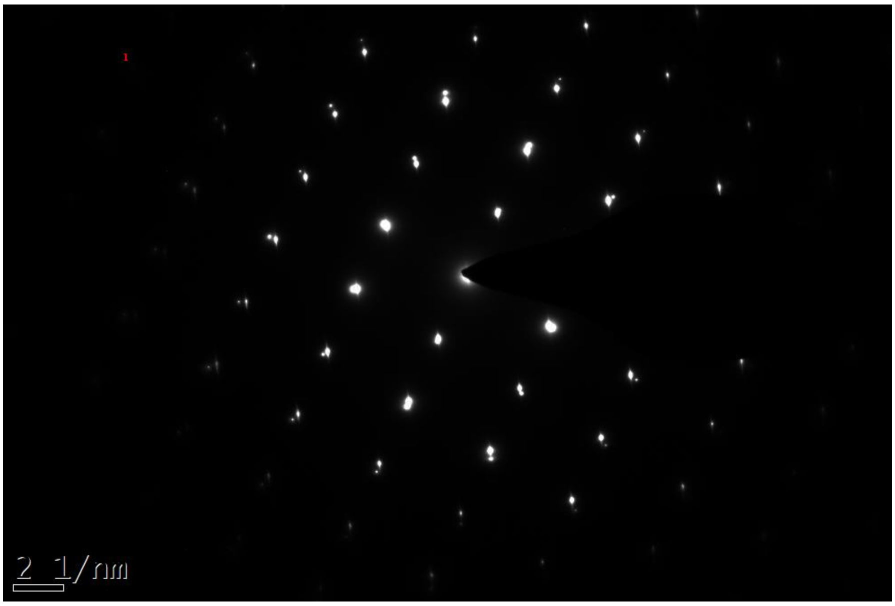

Figure S1. TEM images of the sample grown with Air as the carrier gas. (1) Cross-sectional α-Ga_2O_3/ α-Al_2O_3 interface, (2) diffraction spots of α-Ga_2O_3/α-Al_2O_3.

(1)

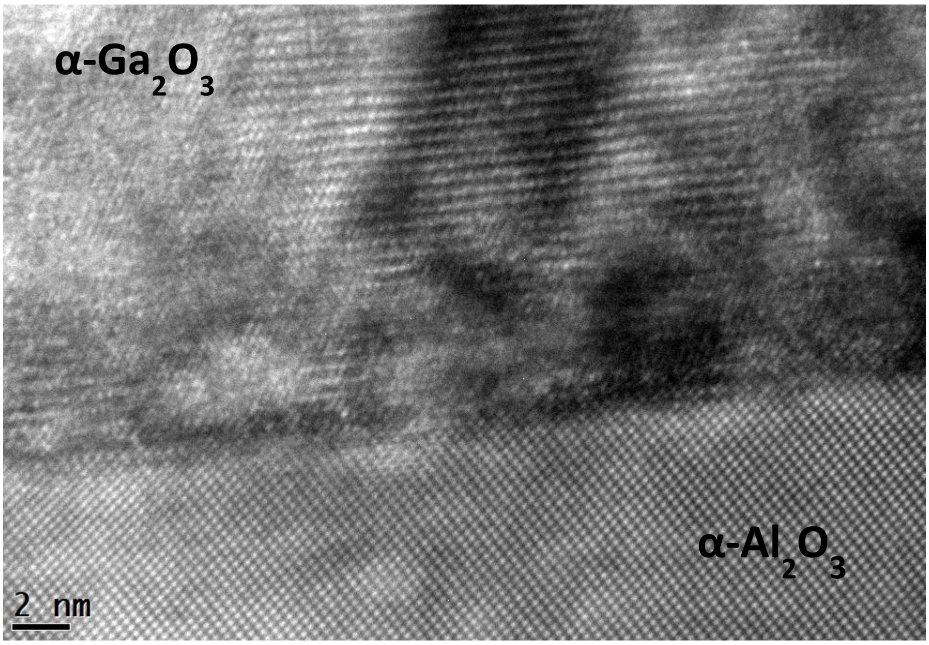

(2)

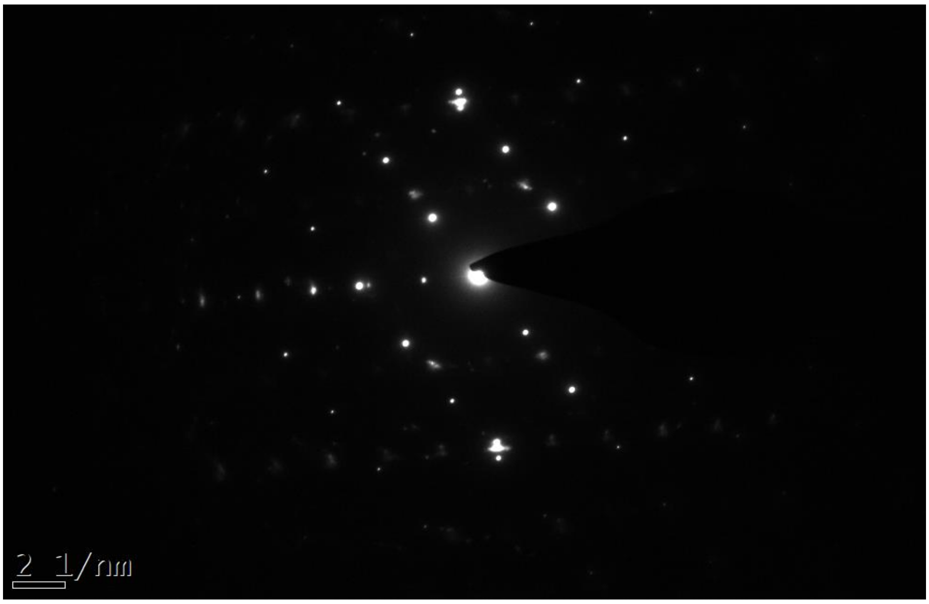

Figure S2. TEM images of the sample grown with N_2 as the carrier gas. (1) Cross-sectional α-Ga_2O_3/ α-Al_2O_3 interface, (2) diffraction spots of α-Ga_2O_3/α-Al_2O_3.

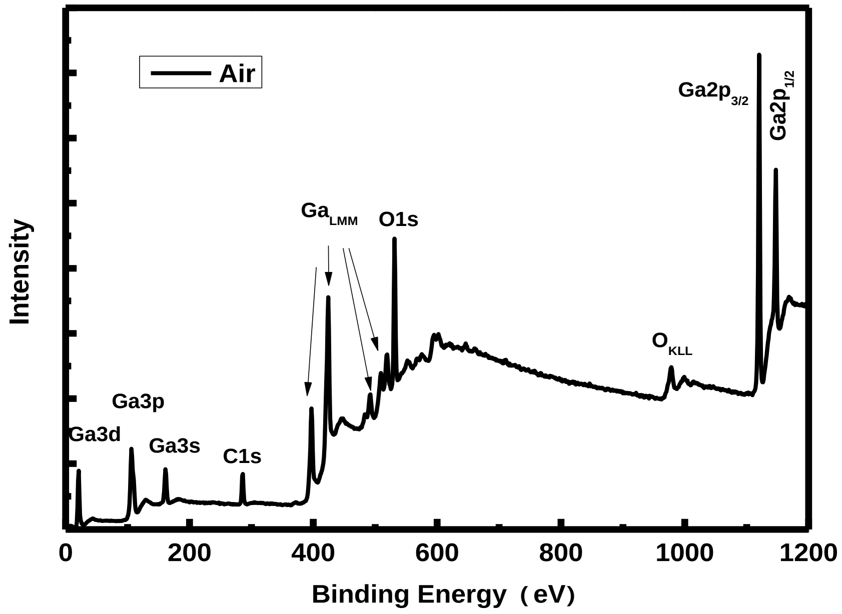

Figure S3. X-ray photoelectron wide spectra for the α-Ga_2O_3 sample grown with Air as the carrier gas.

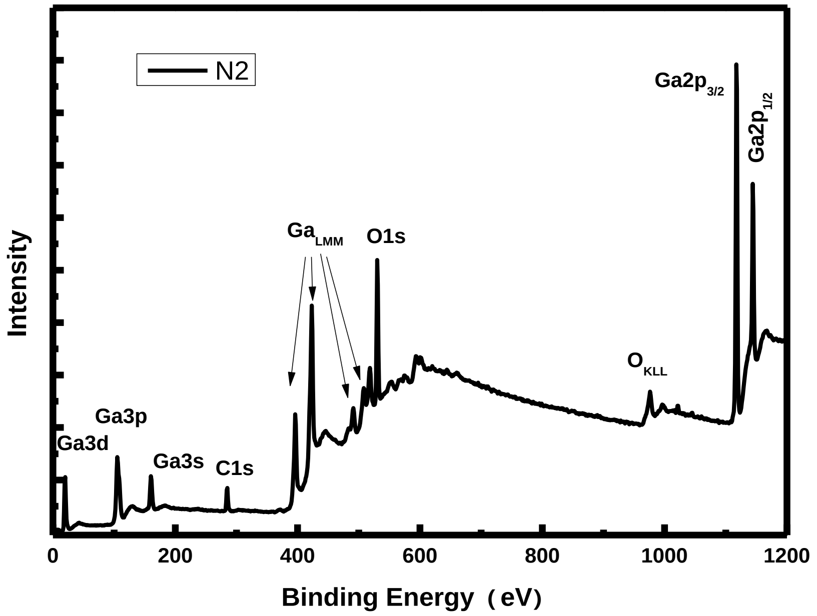

Figure 4. X-ray photoelectron wide spectra for the α-Ga_2O_3 sample grown with N_2 as the carrier gas.

© 2019 by the authors. Licensee MDPI, Basel, Switzerland. This article is an open access article distributed under the terms and conditions of the Creative Commons Attribution (CC BY) license (http://creativecommons.org/licenses/by/4.0/).
